# Factors contributing to male childlessness from a social structural perspective

**DOI:** 10.1093/geroni/igaf122.1130

**Published:** 2025-12-31

**Authors:** Wenqian Xu, Chunyan Kong, Fang Zhao

**Affiliations:** Lund University, Sweden; Fudan University, Shanghai, Shanghai, China (People’s Republic); Fudan University, Shanghai, Shanghai, China (People’s Republic)

## Abstract

Male childlessness in late adulthood is increasingly prevalent in rural China, raising concerns about later-life care and welfare sustainability. While research has examined individual predictors and macro-level conditions, little is known about how these factors interact within the broader structure-agency context. This study, guided by a life course perspective, explores the structural factors shaping childlessness among older men in a rural area of northern China. Drawing on the life stories of 13 childless older men, the study explores how timing, institutional constraints, and life pathway interdependencies affect marriage and childbearing prospects. Structural disadvantages in early life, such as geographic constraints and education exclusion, restricted marriage opportunities. In early adulthood, the hukou system and weak rural welfare reinforced economic hardship and caregiving burdens. By midlife, these disadvantages were compounded by the urban-rural dual structure and entrenched sociocultural norms, further limiting marital prospects. Findings highlight the critical role of place and time in perpetuating socioeconomic disadvantages, as geographic constraints intersect with broader structural factors to shape lifelong trajectories. Addressing childlessness requires policies that support individuals during key life transitions and advocate for institutional reforms, including improved rural welfare, expanded employment opportunities, and reduced urban-rural disparities, to foster equitable conditions for family formation.

